# Incidence Status and Factors Associated With Tyrosine Kinase Inhibitor‐Induced Hypertension in Patients With Renal Cell Carcinoma

**DOI:** 10.1002/cnr2.70219

**Published:** 2025-05-02

**Authors:** Satoru Nakanishi, Keisuke Ikegami, Shungo Imai, Hayato Kizaki, Satoko Hori

**Affiliations:** ^1^ Division of Drug Informatics Keio University Faculty of Pharmacy Tokyo Japan

**Keywords:** hypertension, proton pump inhibitor, renal cell carcinoma, tyrosine kinase inhibitor

## Abstract

**Background:**

Although hypertension is a common side effect of tyrosine kinase inhibitor (TKI) treatment for renal cell carcinoma (RCC), there is limited evidence regarding its occurrence and related risk factors. Preliminary studies suggest that proton pump inhibitors (PPIs) may mitigate the risk of TKI‐induced hypertension; however, their clinical effectiveness remains unclear.

**Aims:**

In this study, we examined the prevalence of TKI‐induced hypertension and the patterns of antihypertensive prescriptions among patients with RCC in Japan. Additionally, we investigated factors associated with TKI‐induced hypertension to assess the potential impact of PPIs.

**Methods and Results:**

Data from patients diagnosed with RCC who were prescribed TKIs between April 2008 and July 2021 were retrospectively gathered from a Japanese administrative database. TKI‐induced hypertension was detected following the diagnosis of hypertension and subsequently the prescription of an antihypertensive agent during TKI therapy. The prescription details for antihypertensive agents were organized in a tabular format. Cox proportional hazards regression analysis was conducted to examine factors contributing to TKI‐induced hypertension. Among the 225 patients analyzed, 36.4% experienced hypertension, and calcium channel blockers were the most prescribed antihypertensive agents. Pre‐existing hypertension was identified as a risk factor for TKI‐induced hypertension, while the concurrent use of PPIs did not show a tendency to reduce the risk of TKI‐induced hypertension.

**Conclusions:**

These findings indicate the importance of blood pressure management in patients with elevated baseline blood pressure.

## 1 Introduction

Tyrosine kinase inhibitors (TKIs) are molecular targeted therapies widely used for various cancers, including renal cell carcinoma (RCC). Multi‐kinase inhibitors used for cancers such as RCC inhibit multiple signaling pathways, including vascular endothelial growth factor (VEGF), thereby suppressing the proliferation of tumor cells. These inhibitors disrupt the tumor cell vascular neogenesis signal and interfere with VEGF signaling in normal endothelial cells, resulting in the common side effect of hypertension. In Japan, the incidence of TKI‐induced hypertension is reported to be higher than that of the international incidence, with hypertension occurring at a proportion of 27.5% [1] and 56% [2] for sorafenib and sunitinib, respectively, as opposed to 17% [3] and 36% [4], respectively. Given that RCC has been reported as a risk factor for TKI‐induced hypertension [5], identifying the background factors of patients with RCC who are particularly prone to developing hypertension is considered clinically pivotal. However, investigations on the occurrence of TKI‐induced hypertension in Japanese patients with RCC have been limited to single‐center studies [6], necessitating the need for large‐scale assessments using data from multiple facilities. Additionally, Proton‐pump inhibitors (PPIs) are frequently used to prevent gastrointestinal complications during treatment. However, since PPIs induce VEGF expression [7], there is a potential for them to lower the risk of TKI‐induced hypertension. Nevertheless, the clinical efficacy of combining PPIs with TKIs to suppress TKI‐induced hypertension remains unclear.

A study suggests that the onset of hypertension following the initiation of TKI treatment serves as an effective biomarker [8], emphasizing the importance of managing blood pressure to maximize treatment effectiveness. While the 2019 hypertension treatment guidelines in Japan recommend diuretics, angiotensin‐converting enzyme inhibitors, angiotensin II receptor blockers, and calcium channel blockers as first‐line antihypertensive agents, information regarding the prescription patterns of antihypertensive agents for patients with RCC is lacking.

Therefore, this study aimed to investigate the occurrence of TKI‐induced hypertension and the prescription patterns of antihypertensive agents for patients with RCC in Japan using a hospital‐based administrative database. Additionally, the study sought to evaluate factors contributing to hypertension onset, including the impact of PPIs.

## 2 Methods

### 2.1 Ethical Considerations

This study was approved by the Ethics Committee of the Keio University Faculty of Pharmacy and adhered to the principles outlined in the Declaration of Helsinki (Approval No: 230511–1). Given that this is a retrospective observational study and the database was pre‐anonymized to prevent individual identification, explicit informed consent was not obtained as reference to Ethical Guidelines for Medical and Biological Research Involving Human Subjects (revised on March 27, 2023) [9].

### 2.2 Data Source

Patient data for individuals diagnosed with RCC, as defined by the International Classification of Diseases, 10th Edition (ICD‐10) codes C64 or C790, were collected from a hospital‐based administrative claims database (Medical Data Vision Co. Ltd., Tokyo, Japan). The data spanned from April 1, 2008 to July 31, 2021. Patients for whom data collection started within 90 days prior to the initial administration of TKIs were excluded. The database compiles insurance claims and data from the Diagnosis Procedure Combination (DPC) system, covering over 40 million patients from more than 460 acute care medical facilities in Japan, with the coverage proportion for DPC‐targeted hospitals approximately 26% [10].

### 2.3 Data Collection

In this study, we collected information on various factors, including age, sex, smoking history, laboratory measurements such as body mass index (BMI), serum albumin (S‐Alb), serum sodium (S‐Na), serum potassium (S‐K), serum creatinine (Scr), aspartate aminotransferase (AST), alanine aminotransferase (ALT), γ‐glutamyl transpeptidase (γ‐GTP), and blood urea nitrogen (BUN). In addition, diagnosis information such as RCC, hypertension (ICD‐10 codes I10, I15‐), and recent diagnoses of liver or kidney diseases within 90 days before the initial administration of TKIs were collected (Table S1). Furthermore, information on drug use, including PPIs, vonoprazan, vitamin D preparations, vitamin E preparations, aspirin, immune checkpoint inhibitors (ICIs), and systemic steroids was recorded (Table S2). Additionally, information on the dosage and duration of administration of the prescribed drugs was collected. We tracked major adverse cardiovascular events (MACE), including myocarditis, pericarditis, Takotsubo cardiomyopathy, atrioventricular block, heart failure, myocardial infarction, and stroke, from the initial TKI prescription date to either the occurrence of TKI‐induced hypertension or two years, whichever came first (Table S3).

### 2.4 Definition of Events

We defined the occurrence of TKI‐induced hypertension as follows: patients were classified based on whether they had pre‐existing hypertension before starting TKI treatment.

- Aggravated Hypertension Group (Cohort 1).

This cohort includes patients with pre‐existing hypertension who increased or added antihypertensive agents within 2 years of initial TKI prescription.

ii New‐Onset Hypertension Group (Cohort 2).

This cohort comprised patients without pre‐existing hypertension who were diagnosed with hypertension for the first time and prescribed antihypertensive agents within 2 years of the initial TKI prescription.

Pre‐existing hypertension was defined as a diagnosis of hypertension within 90 days prior to the first TKI prescription date, with the prescription period of antihypertensive agents including the first TKI prescription date. The TKIs used in this study were sunitinib, sorafenib, axitinib, pazopanib, and cabozantinib, all of which were used for RCC treatment in Japan during the research period. Antihypertensive agents included drugs corresponding to WHO‐Anatomical Therapeutic Chemical (ATC) codes C03 (diuretics), C07 (beta‐blocking agents), C08 (calcium channel blockers), and C09 (drugs acting on the renin‐angiotensin system).

### 2.5 Statistical Analysis

#### 2.5.1 Survey of Antihypertensive Agents Prescription Patterns

A simple tabulation was performed to examine the prescription patterns of the antihypertensive agents associated with TKI‐induced hypertension occurrence.

#### 2.5.2 Analysis of Factors Contributing to TKI‐Induced Hypertension

Clinical characteristics at baseline between the two groups were compared using chi‐square or Fisher's exact tests for categorical variables and Mann–Whitney U tests for continuous variables. The cumulative incidence rates of events during the TKI treatment period were illustrated using Kaplan–Meier curves for patients with baseline hypertension and for those without baseline hypertension, and differences between the two groups were assessed by the log‐rank test. Univariate and multivariate Cox proportional hazards regression analyses were performed. Time‐to‐event was defined as the period from the initial TKI prescription date to either the occurrence of events or two years, whichever came first. Age, pre‐existing hypertension, and BMI, as reported in previous studies [5, 6, 11], were chosen as explanatory variables. Additionally, sex, use of PPIs and systemic steroids, and choice of TKI in the initial prescription were deemed clinically important and included as covariates. Missing values were imputed using the Fully Conditional Specification (FCS) algorithm based on chained equations, resulting in 1000 imputed datasets. SAS 9.4 for Windows (SAS Institute Inc., Cary, North Carolina, USA) was used to conduct the analysis. The significance level for the Cox proportional hazards regression analysis was set at *p* < 0.05.

#### 2.5.3 Sample Size Estimation and Power Calculation

The intended sample size for the Cox proportional hazards regression analysis was approximately 250 cases. While it has been recommended that the events per variable (EPV) be kept at least 10 [12], there has been an ongoing debate regarding EPV, and recent reports [13, 14, 15] support setting the EPV to seven without difficulty. Therefore, 11 explanatory variables were included in the analysis. Thus, the required number of events for Cox proportional hazards regression analysis was estimated to be 77 cases. Based on previous reports [1, 2] and assuming a 30% incidence rate, the required number of TKI prescription cases is 256.

## 3 Results

**FIGURE 1:**
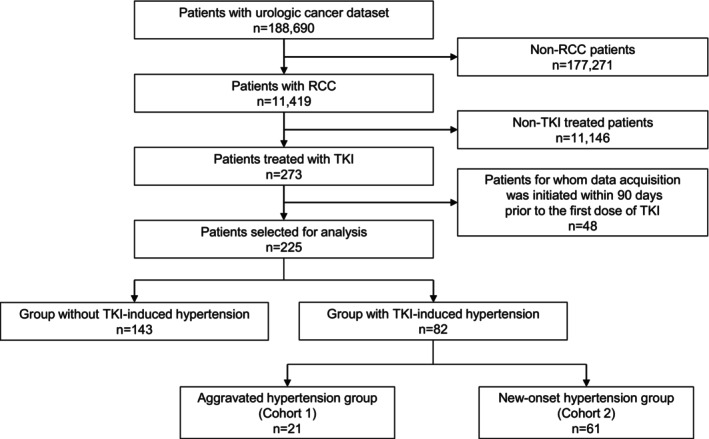
Flow charts of patient extraction. A total of 225 patients were included in the analysis, of whom 82 were identified as having TKI‐induced hypertension. TKI: Tyrosine kinase inhibitor, RCC: Renal cell carcinoma.

Among the 225 cases analyzed, 82 (36.4%) were identified as having TKI‐induced hypertension (Figure 1). Within this group, 21 cases were classified under the Aggravated Hypertension Group (Cohort 1), while 61 cases were classified as belonging to the New‐Onset Hypertension Group (Cohort 2). The median age (interquartile range) was 73.0 (70.0–76.0) years for Cohort 1 and 69.0 (62.0–77.0) years for Cohort 2. Additionally, there were no significant differences in MACE outcomes between the TKI‐induced hypertension group and the non‐occurrence group (Table S4).

### 3.1 Survey of Antihypertensive Agent Prescription Patterns

**FIGURE 2:**
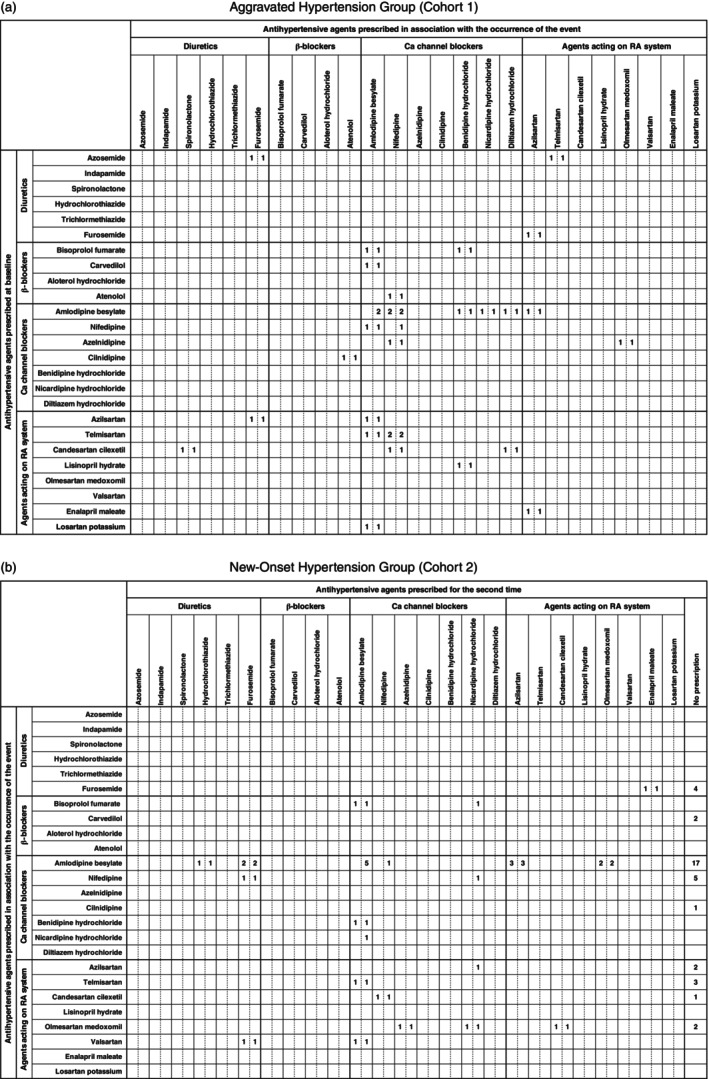
Breakdown of antihypertensive prescriptions. Ca: Calcium, RA: Renin‐angiotensin. The number on the left of each cell indicates the number of cases in which antihypertensive agents were co‐administered. The numbers on the right indicate cases involving co‐administration, switching, or dosage increase. (a) Illustrates the changes in antihypertensive prescriptions for Cohort 1. The vertical axis represents the antihypertensive agents prescribed at baseline, while the horizontal axis shows the drugs prescribed at the time of the event. (b) Depicts the changes in antihypertensive prescriptions for Cohort 2, where the vertical axis indicates the drugs prescribed at the time of the event and the horizontal axis shows the antihypertensive agent prescribed at the second prescription. Tramexamic acid and torbaptan were included in C03‐diuretics but were excluded due to their lack of indication for hypertension.

The breakdown of antihypertensive agent prescriptions is depicted in Figure 2. Following the events, calcium channel blockers were the most prescribed drugs in both cohorts, followed by drugs acting on the renin‐angiotensin system (Figure 2). The median duration from TKI administration to the initiation of antihypertensive agents was 35 (12–122) days in Cohort 1 and 16 (8–65) days in Cohort 2.

Among the 82 cases identified with TKI‐induced hypertension, 41 (50%; Cohort 1: 16 cases, Cohort 2: 25 cases) received combination therapy involving two or more antihypertensive agents. In Cohort 1, among patients initially prescribed calcium channel blockers or drugs affecting the renin‐angiotensin system at baseline, an increase in hypertension prompted the addition of more calcium channel blockers (Figure 2a). Additionally, calcium channel blockers were administered to all patients initially prescribed beta‐blocking agents at baseline. Analysis of drug efficacy classifications revealed a tendency for loop diuretics in the case of diuretics, dihydropyridine‐type for calcium channel blockers, and angiotensin receptor blockers (ARBs) for drugs acting on the renin‐angiotensin system. In Cohort 2, among patients initially prescribed calcium channel blockers, the second prescription was more likely to be a drug other than a beta‐blocking agent. For patients initially prescribed drugs acting on the renin‐angiotensin system, calcium channel blockers were more frequently prescribed as secondary drugs (Figure 2b). In both cohorts, beta‐blocking agents were not prescribed as secondary drugs for patients who received an initial prescription for any antihypertensive agent. The breakdown of the drug efficacy classifications showed a trend similar to that of Cohort 1, and no prescriptions for ACE inhibitors were observed in Cohort 2 following the events.

### 3.2 Analysis of Factors Contributing to TKI‐Induced Hypertension

Table 1 presents the patient characteristics for the TKI‐induced hypertension occurrence group (82 cases) and the non‐occurrence group (143 cases).

**TABLE 1:** Patient demographics.

Values	Without TKI‐induced hypertension *n* = 143	TKI‐induced hypertension *n* = 82	*p*
Sex, *n* (%)			
Male	121 (84.6)	59 (72.0)	0.022
Female	22 (15.4)	23 (28.0)	
Age (years), med (IQR)	71.0 (63.0–77.0)	71.0 (64.0–77.0)	0.836
Smoking history, *n* (%)	41 (28.7)	28 (34.1)	0.578
Baseline hypertension, *n* (%)	26 (18.2)	21 (25.6)	0.187
Initial TKI used, *n* (%)			
Sunitinib	60 (42.0)	29 (35.4)	0.330
Sorafenib	18 (12.6)	8 (9.8)	0.523
Axitinib	19 (13.3)	18 (22.0)	0.092
Pazopanib	46 (32.2)	26 (31.7)	0.943
Cabozantinib	0 (0)	1 (1.2)	0.364^a^
Laboratory values, med (IQR)			
BMI (kg/m^2^)	23.2 (21.3–25.0)	23.8 (20.9–25.3)	0.432
S‐Alb (g/dL)	3.7 (2.8–4.2)	4.0 (3.9–4.3)	0.224
S‐Na (mEq/L)	139.0 (136.5–141.0)	140.2 (139.0–140.7)	0.457
S‐K (mEq/L)	4.7 (4.3–5.0)	4.3 (4.0–4.7)	0.236
Scr (mg/dL)	1.0 (0.8–1.4)	1.1 (0.7–1.6)	0.746
AST (U/L)	21 (15–29)	23 (21–25)	0.816
ALT (U/L)	19.5 (9.5–28.0)	17.5 (16.5–19.5)	0.926
γ‐GTP (U/L)	52.5 (31.0–93.0)	28.5 (21.0–39.0)	0.098
eGFR (mL/min/1.73 m^2^)	52.2 (39.0–60.0)	58.1 (33.5–81.4)	0.746
BUN (mg/dL)	20.6 (16.5–24.0)	19.8 (14.0–25.6)	0.816
Concomitant agents, *n* (%)			
PPI	24 (16.8)	17 (20.7)	0.460
Vonoprazan	4 (2.8)	5 (6.1)	0.292^a^
Vitamin D supplement	4 (2.8)	3 (3.7)	0.708^a^
Vitamin E supplement	0 (0)	0 (0)	—
Aspirin	6 (4.2)	4 (4.9)	1.000^a^
ICI	53 (37.1)	30 (36.6)	0.943
Steroid	55 (38.5)	42 (51.2)	0.063
Comorbidities, *n* (%)			
Renal disease	40 (28.0)	16 (19.5)	0.158
Liver disease	21 (14.7)	8 (9.8)	0.288

*Note:* Smoking history and laboratory data had missing data, with respective missing rates of 43.5% and 91.4%–92.2% (BMI had a missing rate of 39.7%).

Abbreviations: BMI: Body Mass Index, S‐Alb: serum albumin, S‐Na: serum sodium, S‐K: serum potassium, Scr: serum creatinine, AST: aspartate aminotransferase, ALT: alanine aminotransferase, γ‐GTP: γ‐glutamyl transpeptidase, eGFR: estimated Glomerular Filtration Rate, BUN: Blood Urea Nitrogen, PPI: Proton Pump Inhibitor, ICI: immune checkpoint inhibitor.

^a^ Fisher's exact tests.

**FIGURE 3:**
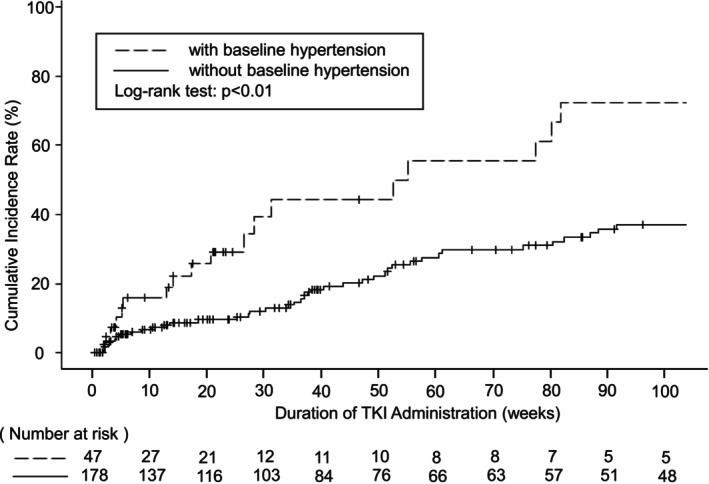
Cumulative incidence rate of TKI‐induced hypertension. Kaplan–Meier curve representing the time from the initial TKI prescription to the occurrence of the event. The dashed line denotes patients with hypertension at baseline, whereas the solid line represents those without hypertension at baseline. The log‐rank test revealed a *p*‐value of < 0.01.

The overall median time to occurrence across both groups was 18 (8–83) days. Among patients without baseline hypertension, the incidence rate was significantly higher than among those with baseline hypertension (Figure 3).

In the multivariate Cox proportional hazards regression analysis (Table 2), pre‐existing hypertension was identified as a risk factor, but no significant differences were observed among the other variables.

**TABLE 2:** Cox proportional hazards regression analysis.

Values	Hazard ratio (95% CI)	*p*	Adjusted hazard ratio (95% CI)	*p*
Sex (female)	1.56 (0.99–2.45)	0.057	0.801 (0.43–1.48)	0.480
Age^a^	1.02 (1.00–1.03)	0.078	1.01 (0.99–1.03)	0.413
Baseline hypertension	0.715 (0.47–1.10)	0.124	1.91 (1.05–3.48)	0.035
BMI^a^	0.967 (0.91–1.03)	0.276	0.933 (0.87–1.00)	0.060
PPI	0.915 (0.59–1.42)	0.691	0.900 (0.47–1.74)	0.754
Initial TKI used				
Sunitinib	1.08 (0.77–1.51)	0.651	1.02 (0.61–1.71)	0.935
Sorafenib	0.725 (0.44–1.19)	0.203	1.16 (0.55–2.47)	0.693
Axitinib	0.899 (0.55–1.46)	0.668	1.24 (0.59–2.61)	0.571
Cabozantinib	—	—	—	—
Pazopanib (Reference)	1.09 (0.77–1.55)	0.624	Reference	—
Steroid	1.22 (0.87–1.71)	0.245	0.809 (0.52–1.26)	0.348

Abbreviations: BMI: Body Mass Index, PPI: Proton Pump Inhibitor, ICI: Immune checkpoint inhibitor.

^a^ Hazard ratio indicates the change in each unit increase.

## 4 Discussion

This study utilized a medical information database to elucidate the occurrence of TKI‐induced hypertension, antihypertensive agent prescription patterns, and the factors contributing to hypertension in patients with RCC, a topic that has not been sufficiently investigated to date. The validity of defining event occurrence is crucial when utilizing medical information databases. The proportion of events in this study was 36.4%, which closely matched the previously reported 40.0% [6]. Additionally, the median number of days from the initiation of TKI prescription to the prescription of antihypertensive agents was 35 (12–122) days for Cohort 1, 16 (8–65) days for Cohort 2, and 18 (8–83) days for both groups. This duration is consistent with previous studies on TKIs, where the median duration ranged from 29 (14–68) days [11], despite the difference in cancer types. Hence, the definition of TKI‐induced hypertension occurrence based on the diagnosis of hypertension and the prescription of antihypertensive agents was deemed to have a certain level of validity.

Calcium channel blockers were the most prescribed antihypertensive drugs in both cohorts. The prescription of two or more antihypertensive agents was confirmed in 50.0% of the patients who experienced TKI‐induced hypertension. Previous research suggests that combining different antihypertensive agents in small amounts may result in better blood pressure control compared to increasing the dosage of a single antihypertensive agent when monotherapy is insufficient [16, 17]. This finding suggests that similar clinical judgments are applied to the management of TKI‐induced hypertension. Even among patients in the new‐onset hypertension group without pre‐existing hypertension (Cohort 2), 25 out of 61 cases required the use of combination antihypertensive agents. This result emphasizes the importance of blood pressure management in TKI treatment for patients with RCC.

Pre‐existing hypertension was identified as a risk factor using the Cox proportional hazards regression analysis, consistent with findings from previous studies [6, 11]. Additionally, concomitant use of PPIs did not show a tendency to reduce the risk of TKI‐induced hypertension. While PPIs are widely used to prevent adverse effects of TKI therapy, their concurrent use has been suggested to affect survival outcomes negatively [18, 19]. Therefore, the appropriateness of concomitant PPI use requires further evaluation from various perspectives.

In interpreting these findings, it is important to recognize that the complexity of TKI regimens in clinical practice limited our ability to track treatment switching and determine which TKI was being used at the time of each event. Although we set a 90‐day lookback period, we were unable to capture TKI prescriptions initiated before that interval or before the start of data collection, further restricting the completeness of our treatment history data. Defining TKI‐induced hypertension by the initiation or intensification of antihypertensive therapy may also have led to an underestimation of its true incidence since mild or transient blood pressure elevations would be unrecognized if no medication was prescribed. This could help explain why our observed incidence of TKI‐induced hypertension was lower than that reported in some prospective trials [2, 20]. In patients without a prior history of hypertension, a few clinicians may have initiated antihypertensive agents prophylactically rather than in response to confirmed elevated blood pressure, yet the absence of longitudinal blood pressure data made it impossible to distinguish true hypertension management from purely preventive measures. Additionally, because mortality and treatment discontinuation were not thoroughly recorded, differences in survival outcomes or competing risks remain unclear. Although our reliance on prescription data and relatively small sample size limit any definitive conclusions about the potential association between PPI use and TKI‐induced hypertension, the findings offer plausible evidence and highlight the need for further research on the clinical implications of PPI co‐administration in this setting.

Despite certain limitations, this study represents the first multi‐institutional, real‐world observational study providing insights into TKI‐induced hypertension in Japanese patients with RCC. Our findings contribute to validating existing knowledge and guiding prospective research, underscoring the necessity for future studies that incorporate broader data sources, clinical laboratory values, comorbidities, and concurrent therapies to extend these insights.

## 5 Conclusions

The prevalence of TKI‐induced hypertension in patients with RCC is 36.4%, with calcium channel blockers being the most frequently prescribed antihypertensive agents in response to this occurrence. Moreover, pre‐existing hypertension has been identified as a risk factor for TKI‐induced hypertension, emphasizing the importance of blood pressure management in patients with elevated baseline blood pressure. However, the study did not find conclusive evidence to support the inhibitory effect of PPIs co‐administration on TKI‐induced hypertension.

## Author Contributions

**Satoru Nakanishi:** conceptualization (lead), data curation (equal), formal analysis (lead), investigation (lead), methodology (lead), validation (lead), visualization (lead), writing – original draft (lead), writing – review and editing (lead). **Keisuke Ikegami:** conceptualization (equal), data curation (equal), methodology (equal), writing – review and editing (equal). **Shungo Imai:** conceptualization (equal), methodology (equal), writing – review and editing (equal). **Hayato Kizaki:** project administration (equal), writing – review and editing (equal). **Satoko Hori:** conceptualization (equal), funding acquisition (lead), project administration (lead), supervision (lead), writing – review and editing (equal).

## Conflicts of Interest

The authors declare no conflicts of interest.

## Supporting information


**Table S1.** Comorbidities and ICD‐10 codes.
**Table S2.** Concomitant medications and their ATC codes.
**Table S3.** MACEs and ICD‐10 codes.
**Table S4.** Number of major adverse cardiovascular events based on MACEs.

## Data Availability

Data analyzed in this study from the Medical Data Vision database, which is anonymized and commercially available data.
