# Supplementary material for: Incidence Status and Factors Associated With Tyrosine Kinase Inhibitor‐Induced Hypertension in Patients With Renal Cell Carcinoma
Source: Cancer Rep (Hoboken). 2025 May 2;8(5):e70219. doi: 10.1002/cnr2.70219 (PMC12046976; doi:10.1002/cnr2.70219)
Supplement: Supplementary file 1 — Table S1. Comorbidities and ICD‐10 codes. Table S2. Concomitant medications and their ATC codes. Table S3. MACEs and ICD‐10 codes. Table S4. Number of major adverse cardiovascular events based on MACEs. [file CNR2-8-e70219-s001.docx]

sTable 1. Comorbidities and ICD-10 codes

| Comorbidity | ICD-10 codes |
| --- | --- |
| Liver diseases | K721, K729, K769 |
| Kidney diseases | N181, N182, N183, N184, N185, N189, N19, N289 |

We identified patients diagnosed with conditions corresponding to the above codes and excluded cases in which diagnoses other than liver or kidney impairment were suspected. (Liver disease: hepatic encephalopathy; kidney disease: post-renal urinary retention, renal anemia, and uremia). Patients with chronic diseases were included if they were diagnosed before the initial administration of TKIs.

sTable 2. Concomitant medications and their ATC codes

| Concomitant medication | ATC codes |
| --- | --- |
| PPIs | A02B plus text code (esomeprazole, omeprazole, rabeprazole, lansoprazole) |
| Vonoprazan | A02B plus text code (vonoprazan) |
| Vitamin D preparation | A11C plus text code (alfacalcidol, calcitriol), text code (erdecalcitol, falecalcitriol, maxacalcitol)* |
| Vitamin E preparation | A11 plus text code (tocopherol) |
| Aspirin | B01 plus text code (aspirin), N02 plus text code (aspirin) |
| ICIs | L01 plus text code (nivolumab, ipilimumab, pembrolizumab, avelumab) |
| Steroid | H02A |

* Erdecalcitol, falecalcitriol, and maxacalcitol are not listed in the WHO-ATC code; therefore, they were extracted using text codes. PPIs: proton pump inhibitors ICIs: immune checkpoint inhibitors; (ATC) codes: Anatomical Therapeutic Chemical Classification System

sTable 3. MACEs and ICD-10 codes

| MACEs | ICD-10 codes |
| --- | --- |
| Myocarditis | I40, I41 |
| Pericarditis | I30, I31, I32 |
| Takotsubo cardiomyopathy | I518 |
| Atrioventricular block | I44, I45 |
| Heart failure | I500, I501, I509, I110 |
| Myocardial infarction | I210, I211, I212, I213, I214, I219 |
| Stroke | I600, I601, I602, I603, I604, I605, I606, I607, I608, I609, I610, I611, I613, I614, I615, I616, I619, I629, I630, I631, I632, I633, I634, I635, I636, I638, I639, G459 |

MACE: Major Adverse Cardiovascular Events, ICD-10: International Classification of Diseases, 10th Edition

sTable 4. Number of major adverse cardiovascular events based on MACEs

| Values | Without  TKI-Induced hypertension  n=143 | TKI-induced hypertension  n=82 |
| --- | --- | --- |
| MACEs* | 15 (10.5) | 11 (13.4) |
| Myocarditis | 0 (0) | 0 (0) |
| Pericarditis | 1 (0.7) | 0 (0) |
| Takotsubo cardiomyopathy | 0 (0) | 0 (0) |
| Atrioventricular block | 0 (0) | 0 (0) |
| Heart failure | 8 (5.6) | 10 (12.2) |
| Myocardial infarction | 2 (1.4) | 1 (1.2) |
| Stroke | 4 (2.8) | 0 (0) |

MACE: Major Adverse Cardiovascular Events. Data are expressed as number (percentage). *p=0.509
